# SEanalysis 2.0: a comprehensive super-enhancer regulatory network analysis tool for human and mouse

**DOI:** 10.1093/nar/gkad408

**Published:** 2023-05-17

**Authors:** Feng-Cui Qian, Li-Wei Zhou, Yan-Yu Li, Zheng-Min Yu, Li-Dong Li, Yue-Zhu Wang, Ming-Cong Xu, Qiu-Yu Wang, Chun-Quan Li

**Affiliations:** The First Affiliated Hospital, Cardiovascular Lab of Big Data and Imaging Artificial Intelligence, Hengyang Medical School, University of South China, Hengyang, Hunan 421001, China; The First Affiliated Hospital, Institute of Cardiovascular Disease, Hengyang Medical School, University of South China, Hengyang, Hunan 421001, China; National Health Commission Key Laboratory of Birth Defect Research and Prevention, Hunan Provincial Maternal and Child Health Care Hospital, Changsha, Hunan 410008, China; School of Computer, University of South China, Hengyang, Hunan 421001, China; Insititute of Biochemistry and Molecular Biology, Hengyang Medical College, University of South China, Hengyang, Hunan 421001, China; The First Affiliated Hospital, Department of Cardiology, Hengyang Medical School, University of South China, Hengyang, China; School of Medical Informatics, Daqing Campus, Harbin Medical University, Daqing 163319, China; School of Medical Informatics, Daqing Campus, Harbin Medical University, Daqing 163319, China; The First Affiliated Hospital, Cardiovascular Lab of Big Data and Imaging Artificial Intelligence, Hengyang Medical School, University of South China, Hengyang, Hunan 421001, China; School of Computer, University of South China, Hengyang, Hunan 421001, China; School of Medical Informatics, Daqing Campus, Harbin Medical University, Daqing 163319, China; The First Affiliated Hospital, Cardiovascular Lab of Big Data and Imaging Artificial Intelligence, Hengyang Medical School, University of South China, Hengyang, Hunan 421001, China; The First Affiliated Hospital, Cardiovascular Lab of Big Data and Imaging Artificial Intelligence, Hengyang Medical School, University of South China, Hengyang, Hunan 421001, China; The First Affiliated Hospital, Institute of Cardiovascular Disease, Hengyang Medical School, University of South China, Hengyang, Hunan 421001, China; National Health Commission Key Laboratory of Birth Defect Research and Prevention, Hunan Provincial Maternal and Child Health Care Hospital, Changsha, Hunan 410008, China; School of Computer, University of South China, Hengyang, Hunan 421001, China; Insititute of Biochemistry and Molecular Biology, Hengyang Medical College, University of South China, Hengyang, Hunan 421001, China; The First Affiliated Hospital, Department of Cardiology, Hengyang Medical School, University of South China, Hengyang, China; The First Affiliated Hospital, Cardiovascular Lab of Big Data and Imaging Artificial Intelligence, Hengyang Medical School, University of South China, Hengyang, Hunan 421001, China; The First Affiliated Hospital, Institute of Cardiovascular Disease, Hengyang Medical School, University of South China, Hengyang, Hunan 421001, China; National Health Commission Key Laboratory of Birth Defect Research and Prevention, Hunan Provincial Maternal and Child Health Care Hospital, Changsha, Hunan 410008, China; School of Computer, University of South China, Hengyang, Hunan 421001, China; Insititute of Biochemistry and Molecular Biology, Hengyang Medical College, University of South China, Hengyang, Hunan 421001, China; The First Affiliated Hospital, Department of Cardiology, Hengyang Medical School, University of South China, Hengyang, China

## Abstract

Super-enhancers (SEs) play an essential regulatory role in various biological processes and diseases through their specific interaction with transcription factors (TFs). Here, we present the release of SEanalysis 2.0 (http://licpathway.net/SEanalysis), an updated version of the SEanalysis web server for the comprehensive analyses of transcriptional regulatory networks formed by SEs, pathways, TFs, and genes. The current version added mouse SEs and further expanded the scale of human SEs, documenting 1 167 518 human SEs from 1739 samples and 550 226 mouse SEs from 931 samples. The SE-related samples in SEanalysis 2.0 were more than five times that in version 1.0, which significantly improved the ability of original SE-related network analyses (‘pathway downstream analysis’, ‘upstream regulatory analysis’ and ‘genomic region annotation’) for understanding context-specific gene regulation. Furthermore, we designed two novel analysis models, ‘TF regulatory analysis’ and ‘Sample comparative analysis’ for supporting more comprehensive analyses of SE regulatory networks driven by TFs. Further, the risk SNPs were annotated to the SE regions to provide potential SE-related disease/trait information. Hence, we believe that SEanalysis 2.0 has significantly expanded the data and analytical capabilities of SEs, which helps researchers in an in-depth understanding of the regulatory mechanisms of SEs.

## Graphical Abstract

**Graphical Abstract:**
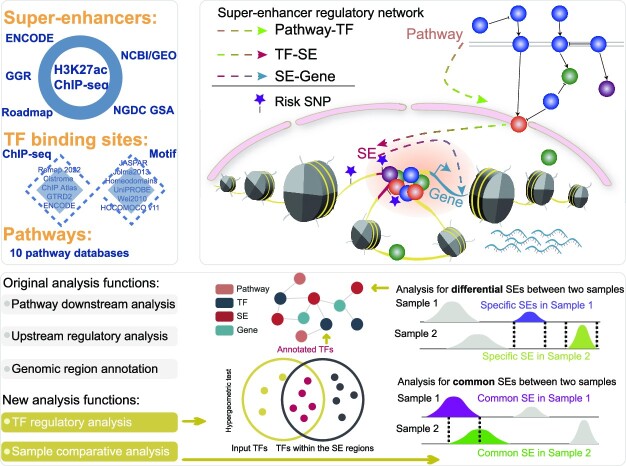

## INTRODUCTION

Super-enhancers (SEs) as DNA regulatory elements have the superior ability to recruit large amounts of transcription factors (TFs) binding and further prominently regulating the expression of key genes that control cell identity (1). These TFs are usually regulated by key signaling pathways that play a crucial role in cell development. Studies have revealed that the signaling pathways and their terminal TFs can influence disease progression and cell lineage development by remodelling the SE landscape, such as changing the distribution of SEs (2–4). For example, by comparing SE profiles before and after *PRRX1* deletion, TF *PRRX1* was found to change the distribution of SEs via TGF-β signaling to orchestrate the functional drift of fibroblasts into the myofibroblastic phenotype (3). The Wnt signaling pathway can activate the expression of a canonical cancer driver *MYC* by collaborating with oncogenic SEs in colon cancer (5). An emerging study found that a group of patients with mutations of SE of the *TAL1* oncogene exhibited a poor prognosis regardless of the level of oncogene dysregulation (6). This study demonstrated that the mechanism of SE-mediated oncogene dysregulation was critical for clinically distinct patient subgroups, and further emphasized the future of SE targeting therapy (6). Obviously, the complex networks formed by the functional interplay among pathways, TFs, SEs and genes are particularly critical in the study of regulatory mechanisms of many biological processes. Thus, we developed SEanalysis (7) for downstream and upstream regulatory network analysis of SEs in 2019.

Exploring SEs has gradually revealed their mechanism of action in complex networks. A large number of studies confirmed that TFs played a crucial role in the regulation of SEs. For example, the hormone-stimulated glucocorticoid receptor (*GR*) can bind to the upstream super-enhancer region of oncogene *DDIT4 to* promote the interaction between *GR* and *DDIT4* through the formation of a chromatin loop (8). Therefore, revealing the regulatory mechanism of TFs in SE-associated regulatory networks by dissecting pathway–TF–SE–gene interactions in certain biological processes is of great significance. Further, studies showed that dynamically changing SEs influenced by SNPs were often key factors in promoting cell state changes compared with other SEs. For example, a B-cell-restricted SE, which was in contact with the SNP locus associated with systemic lupus erythematosus (SLE) and was mediated by *STAT3*, was associated with B-cell deregulation in SLE (9). Hence, several studies focused on the clues of marker gene dysregulation, especially the upstream SEs and their TFs and SNPs.

**Figure 1.**
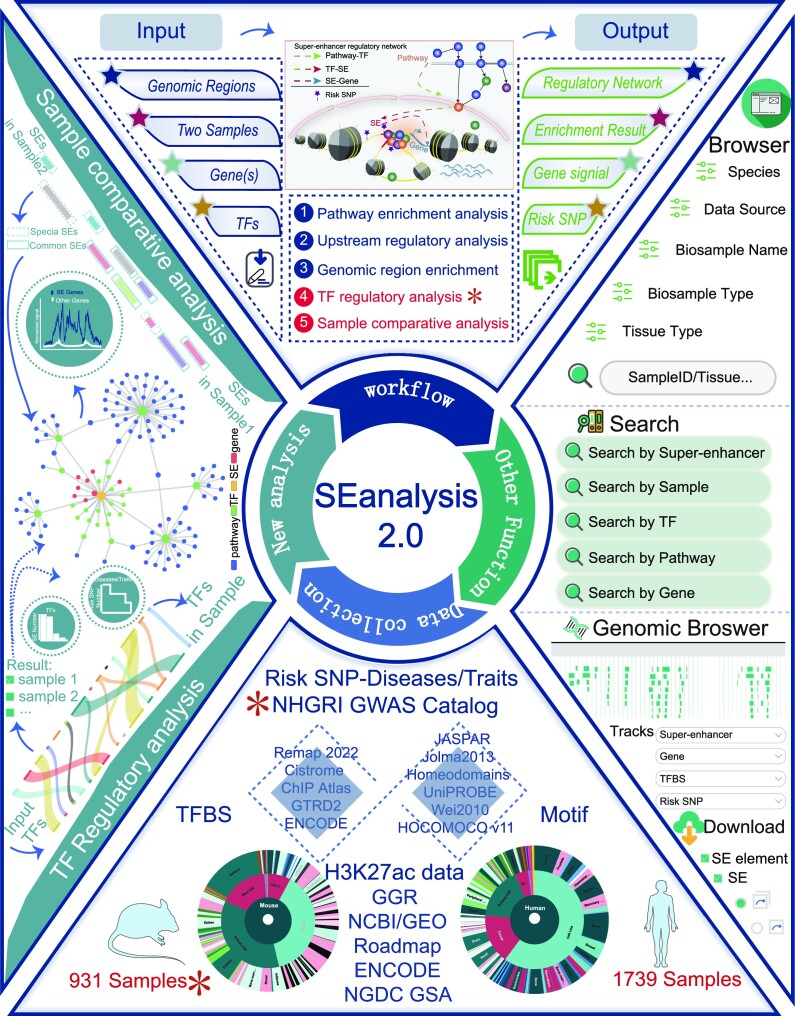
SEanalysis 2.0 function and construction. SEanalysis 2.0 constructs super-enhancer-associated regulatory network formed by SEs, TFs, pathways and genes. It supports five SE-associated analyses: (i) pathway downstream analysis; (ii) upstream regulatory analysis; (iii) genomic region annotation; (iv) TF regulatory analysis and (v) sample comparative analysis. SEanalysis 2.0 also helps browse, search, download and visualize SEs.

To further meet the need of researchers and elucidate the regulatory mechanisms of SEs-associated network, we developed SEanalysis 2.0, which significantly expanded the data and analytical capabilities of SEs (Figure 1). Currently, SEanalysis 2.0 documents 1 717 744 SEs from 2670 samples, including 1739 human samples and 931 mouse samples. Importantly, we added two new analyses (‘TF regulatory analysis’ and ‘Sample comparative analysis’), and significantly extended three original analysis functions for understanding context-specific gene regulation. Furthermore, we provided annotation information on risk SNPs to link SEs with diseases/traits. Hence, SEanalysis 2.0 not only expanded species and large-scale data, but also facilitated more comprehensive analyses, which might further promote the understanding of the biological mechanisms of epigenomic network regulation.

## DESCRIPTION OF THE WEB SERVER

### Data expansion

#### Super-enhancers

Compared with SEanalysis 1.0, SEanalysis 2.0 provided significant data improvement. We added 931 mouse SE sets and extended 1198 human SE sets from the H3K27ac ChIP-seq data of the SEdb 2.0 database developed by our group. Currently, the SE-related ChIP-seq samples in SEanalysis 2.0 are more than five times than those in SEanalysis 1.0 (Supplementary Table S1). Specifically, the raw H3K27ac ChIP-seq data were collected from the NCBI GEO/SRA (10), ENCODE (11), Roadmap (11,12), Genomics of Gene Regulation Project (GGR) (11) and National Genomics Data Center Genome Sequence Archive (NGDC GSA) (13,14). In particular, the raw sequencing reads were processed via optimized SE identification workflow (Bowtie2-MACS2-ROSE) and the reference genomes were updated to hg38 and mm10. Currently, SEanalysis 2.0 includes 1 167 518 human SEs from 1739 samples involving ∼180 cells/tissues and 550 226 mouse SEs from 931 samples involving ∼110 cells/tissues.

#### SE-target genes

In SEanalysis 1.0, four different strategies were used to annotate SE-associated genes: closest active genes (15), overlapping genes, proximal genes and the closest genes (16). Besides these original strategies, SEanalysis2.0 also provided two new target gene identification strategies named ‘JEME’ and ‘Prestige’. The ‘JEME’ strategy constructed enhancer–target networks in 935 samples described in (17). The ‘Prestige’ strategy proposed in a previous study (18) connected the enhancers with their gene targets in 13 samples. We obtained these enhancer-gene relationships to assign target genes to our SEs. The histone modifications were strongly associated with gene expression, such as active promoters were enriched by H3K27ac (19–21). Thus, we calculated the H3K27ac signal density in gene regions as the activity scores of each gene using deepTools (Supplementary Figure S1A, Supplementary Material) (22). Briefly, we first normalized H3K27ac ChIP-seq data with different sequencing depths using deepTools bamCoverage with parameter ‘–normalizeUsing RPKM’ to allow for unbiased comparisons of signal intensities. Then, the deepTools computeMatrix was used for extracting the activity scores for each gene with the following parameters ‘scale-regions -p 10 –beforeRegionStartLength 3000 –regionBodyLength 5000 –afterRegionStartLength 3000 –skipZeros’.

#### Transcription factor data

We further extended the TF ChIP-seq data and the motif data, which were rapidly accumulated in recent years, to obtain a more comprehensive TF-SE relationship. For the TF ChIP-seq data, we looked at the corresponding databases, including ReMap 2022 (23), Cistrome (24), ChIP-Atlas 2021 (25), GTRD (26) and ENCODE (11), all of which added large amounts of high-quality TF ChIP-seq data to their updated versions. We obtained 10 710 human TF ChIP-seq samples and 1051 mouse TF ChIP-seq samples involving 1468 human TFs and 446 mouse TFs. Then, we identified TF-SE regulatory relationships based on the TF-binding sites in constituent enhancers of SE regions of corresponding cell/tissue types using BEDTools (v2.25.0) (27). We also predicted TF-SE regulatory relationships based on motif scanning using FIMO software with a *P*-value threshold of 1e–5 (28,29). For motif data, we collected 3680 human TF motifs of 869 TFs from HOCOMOCO v11 (30), JASPAR (31), Jolma2013 (32), Homeodomains (33), UniPROBE (34), and Wei2010 (35). HOCOMOCO is a new data source added in SEanalysis 2.0, which is a widely used motif source. Further, we added 742 mouse TF motifs of 568 TFs from HOCOMOCO v11 and UniPROBE. In order to reduce the limitations of motif-based prediction, we supported stricter FIMO threshold settings and calculated the number of TF binding sites in SE region to help filter TF-SE relationships.

#### Risk SNP annotation

We collected risk SNP information from the GWAS Catalog (36), which contained risk SNP locations, rsID and related diseases/traits. Then, we filtered risk SNPs lacking location information and obtained 449 062 risk SNPs related to diseases/traits. Finally, we used BEDTools to annotate these risk SNPs to the SE regions when the SNP locations overlapped with the constituent enhancers of SE regions (Supplementary Material, Figure S1B). Furthermore, we further calculated the number of risk SNPs related to each disease/trait for each sample. These annotation details of risk SNPs were displayed on the web server using interactive charts.

#### Sequence conservation

We first obtained phastCons scores for human and mouse sequences from the UCSC browser, which were measured based on multiple alignments of 99 vertebrate genomes to the human genome and 59 vertebrate genomes to the mouse genome, respectively (Supplementary Material). Then, we used the bigwigAverageOverBed tool to calculate the conservation of each SE (37).

### Enhanced functions in SEanalysis 2.0

In SEanalysis 2.0, the H3K27ac samples were significantly expanded. Notably, SEanalysis 2.0 included 1739 human samples and 931 mouse samples. Human SEs were increased from about 330 000 to 1 167 518 and 550 226 mouse SEs were added. Meanwhile, the human TFs increased from 1044 to 1700, and 755 mouse TFs were added. With increasing data, the SE-associated regulatory network covered a larger amount of regulatory information about SEs, TFs, potential pathways, and genes. Thus, the original three analysis functions in SEanalysis 1.0 (pathway downstream analysis, upstream regulatory analysis, and genomic region annotation) were significantly improved in SEanalysis 2.0. Furthermore, we added the gene activity score and risk SNP annotation information to the original analyses, thus further improving the interpretability of analysis results in SEanalysis 2.0.

We developed a new analysis function as ‘TF regulatory analysis’ for supporting more comprehensive analyses of SE regulatory networks driven by TFs, so as to further understand the context-specific TF regulation (Supplementary Figure S2A). The ‘TF regulatory analysis’ helped users discover the tissues/cells regulated by TFs of interest through SEs, and further elucidate TF-related functions and potential biological mechanisms in the specific tissue/cell. Specifically, SEanalysis 2.0 first determined a scope of TFs in each sample based on the ‘FIMO Threshold’ with the input of TFs of interest and the setting of the enrichment significance *P*-value, SE-gene linking strategies, and FIMO threshold. Subsequently, we performed the hypergeometric test between the input TFs and TF set of each sample to identify the enriched samples. After the enrichment analysis results were filtered through the pre-set significance *P*-value threshold’, the significantly enriched samples and their information were displayed in the result table. The result table included ‘Sample ID’, ‘Species’, ‘Tissue type’, ‘Biosample name’, ‘Annotated TF’, ‘Annotated TF number’, ‘ALL TF number’, ‘P_value’ and ‘q_value’. Next, the users could further select up to two samples of interest and click on the ‘Submit’ button to obtain the detailed regulatory information and visualization, including regulatory network, risk SNP annotation, gene activity score and statistics information. Among these, the regulatory network is formed by annotated TFs, pathways containing these TFs, SEs bound by TFs, and SE-associated genes. For each sample, the relationships between SEs and annotated TFs were built through ChIP-seq data and motif scanning under a pre-set ‘FIMO Threshold’. SEs and their target genes were linked using ‘SE-Gene linking strategy’. We established pathway-TF relationship if the TF was a component of the pathway. Finally, these relationships were merged to construct regulatory networks. Furthermore, we calculated the number of SEs bound by each annotated TF and displayed it in the histogram. The activity score of genes related to SEs bound by TFs was also visualized. We performed risk SNP annotation for each SE. The number of risk SNPs for each disease/trait associated with SEs was shown in the bar chart.

SEs are usually considered as cell/tissue-specific DNA regulatory elements. We added ‘Sample comparative analysis’ function to explore the roles of differential SEs and common SEs between two samples of interest (Supplementary Figure S2B). The users could select two SE samples of interest filtering by ‘Species’, ‘Tissue Type’ and ‘Sample Name’. Further, the users could choose multiple thresholds, including ‘FIMO Threshold’ and ‘SE-Gene Linking Strategies’. Next, we will compare the genomic regions of SEs between the two selected samples, these non-overlapping regions as specific SEs and overlapping region as common SEs of each sample (Supplementary Figure S2B). The ‘Sample comparison analysis’ returned the detailed regulatory network information of common/specific SEs in the two samples, which will contribute to evaluate the different regulatory roles of these SEs. The output result included the following: (i) the detailed information of the selected SE samples; (ii) the table and visualization of corresponding regulation networks of common/specific SEs in two samples; (iii) the gene activity score of common/specific SE-target genes; (iv) the topology of each node in the network, including degree, closeness, betweenness and page rank and (v) the ratio of risk SNPs within the SE region for each disease/trait.

### Implementation

The current version of the SEanalysis website runs on a Linux-based Tomcat web server 8.5.78 (http://tomcat.apache.org/). All data in this program is stored in the relational database MySQL 5.7.16 (http://www.mysql.com). We built the project using Spring Boot 2.7.0 framework (https://spring.io/projects/spring-boot). The SEanalysis web interface was designed and built using Bootstrap v5.1.3 (https://getbootstrap.com/) and jQuery v3.6.0 (http://jquery.com). We used ECharts (http://echarts.baidu.com) as a graphical visualization framework, and JBrowse (https://jbrowse.org/jb2/) as the genome browser framework. We proposed using a modern web browser that supported the HTML5 standard such as Firefox, Google Chrome and Edge for the best display.

## CASE STUDY

**Figure 2.**
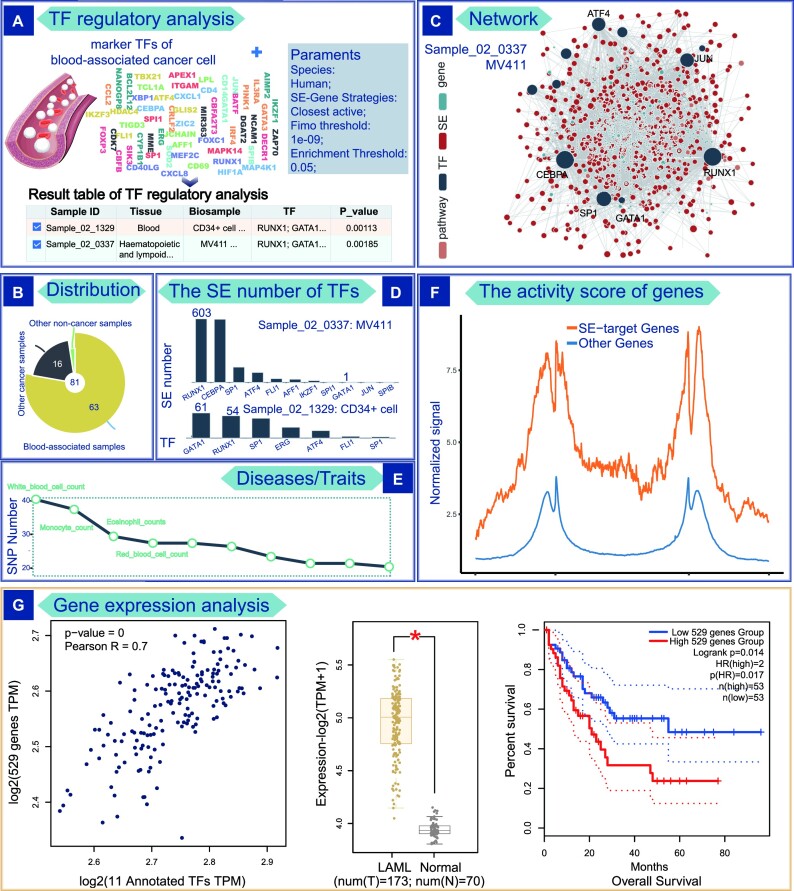
Results of TF regulatory analysis. (**A**) A marker TF list of cancer cells in blood tissue was considered as the input of TF regulatory analysis. The analysis result is displayed in the table. (**B**) Sample distribution. (**C**) Network visualization. (**D**) Number of SEs bound by each TF. (**E**) SE-associated diseases/traits based on risk SNPs. (**F**) Activity score of SE-target genes. (**G**) SE-target genes expression analysis.

To explore the mechanism of action of leukemic cell marker TFs mediated by SEs, we downloaded 58 marker TFs of cancer cells in blood tissue from TF-Marker (38) database as input for the TF regulatory analysis, including *SP1* and *FLI1*, etc. (Figure 2A). The parament settings were as follows: Species: Human; Fimo threshold: 1e–09; Enrichment Threshold: 0.05 and SE-Gene Linking Strategies: Closest active. First, SEanalysis 2.0 performed enrichment analysis based on the input TFs and TFs binding to SEs in each sample after clicking the ‘Analysis’ button. The enrichment analysis results were displayed in the table, including ‘Sample ID’, ‘Species’, ‘Tissue type’, ‘Biosample name’, ‘Annotated TF’, ‘Annotated TF number’, ‘ALL TF number’, ‘P_value’ and ‘q_value’ (Figure 2A). A total of 81 samples were significantly enriched. Among these, 63 (72%) enriched samples were related to blood, bone marrow, haematopoietic, and lymphoid tissues. Further, 16 of the remaining 18 samples were cancer cells/tissues, including breast cancer and renal clear cell adenocarcinoma (Figure 2B). This result suggested that these TFs, the markers of cancer cells, might potentially impact the development and progression of cancer by regulating SEs.

The leukaemia cell sample (Sample_02_0337; MV411; *P* value = 0.00185) and the non-cancer sample (Sample_02_1329; CD34 + cells; *P* value = 0.00113) ranked highly as second and first among all enriched samples (Figure 2A). The number of annotated TFs in the two samples was 11 and 7, respectively. Next, we selected these two samples and clicked the ‘Submit’ button to further explore the regulatory mechanism of TFs. We first constructed the transcriptional regulatory network formed by these annotated TFs in the corresponding sample, including the pathway containing these TFs, SEs bound by these TFs, and SE-related genes (Figure 2C). This network was visualized interactively and could also be viewed in the table. The node size represented its degree in the network. The number of SEs bound by TFs was displayed in a histogram (Figure 2D). We found that most TFs bound more SEs in cancer cells than in non-cancer cells. For example, TF *RUNX1* with the highest degree regulated 606 SEs in the leukaemia cell line MV411 and only 54 SEs in non-cancer cells. In contrast, TF *GATA1* regulated only one SE in MV411 cells, but it regulated 61 SEs in non-cancer cells. Meanwhile, we used limma (39) to obtain the differential expression information based on GEPIA2 (40) tool on acute myeloid leukemia (LAML) data from TCGA. The result showed that *RUNX1* and *GATA1* were significantly differentially expressed genes. *RUNX1* is an over-expressed gene (log_2_(FC): 2.686; *P* value: 4.73e–74), whereas *GATA1* is an under-expressed gene (log_2_(FC): –4.762; *P* value: 5.41e–62). Furthermore, the annotation result of SNP showed that these SE regions tended to have SNPs related to blood cell processes and other traits. We provided a histogram to display the SNP number of each disease/trait in the MV411 sample. Among the top 10 diseases/traits, 7 were related to blood cells, including White_blood_cell_count and Monocyte_count (Figure 2E).

Finally, we analyzed 529 genes associated with SEs occupied by 11 annotated TFs. Notably, these genes exhibited significantly higher activity scores compared with other genes in the leukaemia cell line MV411 (Figure 2F). Next, we further analyzed these SE-target genes in LAML using GEPIA2. As expected, the scatter chart indicated that target genes of SEs were significantly correlated with TFs binding to SEs (Pearson *r* = 0.7, *P* value = 0) (Figure 2G). The box diagram and heatmap showed that the expression levels of these genes were significantly higher in LAML compared with normal samples. More importantly, the survival analysis showed that the high expression of these genes was associated with poor overall survival (Figure 2G).

The aforementioned results indicated that *RUNX1* and other marker TFs caused the abnormal transcription of downstream genes in diseases by regulating SEs, and affected the disease process and survival. At the same time, specific disease/trait-related SNPs played an important role in activating and inhibiting SEs. Consistent with our results, a previous study showed that the SE in intragenic of *RUNX1* was editing-outed will repressed *RUNX1*, further inhibited cell growth and induced death in AML cells expressing *mtRUNX1* (41). This proved the effective analytical ability of SEanalysis 2.0 tools. Furthermore, SEanalysis 2.0 also revealed the novel regulatory mechanism of master TF. Another study showed that *GATA1* maintained the expression of the *KIT* receptor in human erythroid progenitors by binding to a stage-specific SE (42). Several studies further showed that the mutation of *KIT* was significantly associated with a poor prognosis (43). The regulatory mechanism of *GATA1* and SE in leukaemia was still unclear. Our analysis results hinted that the dysregulation of *GATA1* during leukemia progression was likely associated with its upstream SE (Figure 2D).

## SUMMARY

The complex network mediated by SEs and associated TFs underlies lineage identity (44). SEanalysis has been developed to provide SE-associated regulatory network analyses. A large number of recent studies focused on the clinical role of SE-mediated molecular mechanisms affecting oncogene dysregulation. TFs and SNPs play an important role in the action of SEs. They can activate or inhibit the SEs and affect their upstream and downstream regulatory relationships. Meanwhile, increasing evidence indicates that SEs can be considered potential drug targets. To further advance mechanistic research, we developed SEanalysis 2.0 for more comprehensive and flexible analyses for SE-related regulatory networks. SEanalysis 2.0 had two major improvements: data extension and enhanced analysis functions. SEanalysis 2.0 currently offers 1739 human samples and 931 mouse samples, which are at least 5 times as many as in SEanalysis 1.0. Meanwhile, TF ChIP-seq data and motifs are further collected to cover more TFs. We added >600 human TFs and newly added 755 mouse TFs.

The explosion of SEs and TFs has made the regulatory relationship coverage more comprehensive, thus significantly improving the ability of three SE-related network analysis tools in the original version 1.0 (‘pathway downstream analysis’, ‘upstream regulatory analysis’ and ‘genomic region annotation’). We also added two analytical tools: ‘TF regulatory analysis’ and ‘Sample comparative analysis’ for supporting more comprehensive analyses of SE regulatory networks driven by TFs. The urgent need to elucidate the regulatory mechanisms of TFs mediated by SEs promotes the establishment of ‘TF regulatory analysis’ which can facilitate a comprehensive analysis of SE-driven TF regulatory networks. The effectiveness of TF regulatory analysis was supported by the analysis of leukaemia samples. Furthermore, SEs can orchestrate cell type-specific gene regulation. The regulatory network analysis for special/common SEs in two samples is essential for understanding context-specific gene regulation. Thus, we also developed the ‘Sample comparative analysis’ to help interpret cell type-specific regulatory roles of these SEs. The cell type-specific regulation of SEs is often associated with important biological processes and diseases. Accordingly, we further provided the annotation and statistics of risk SNPs, which could link SEs with diseases/traits. The enrichment of risk SNPs in these SE regions could provide information on mechanisms underlying diseases/traits at a cell type-specific level.

Hence, we believed that SEanalysis 2.0 significantly expanded the data and analytical capabilities of SEs. SEanalysis 2.0 helped better explore the key role of SEs in molecular mechanisms underlying disease occurrence and cell biological processes, thus providing a more in-depth understanding of SEs for researchers. Notably, although we have used a large amount of ChIP-seq data to construct SE-TF relationships, ChIP-seq data cannot cover more TFs or cells/tissues. We extended SE-TF relationships using the motif-based method. However, the motif-based method has certain limitations. For example, some TFs with similar motifs are likely to be identified together increasing the number of non-functional hits. Obviously, it is necessary to continue to expand the TF-SE relationships identified from ChIP-seq data. With the accumulation of ChIP-seq data, we will continue to update the SEanalysis.

## DATA AVAILABILITY

SEanalysis 2.0 is freely available without registration or login at http://licpathway.net/SEanalysis.

## Supplementary Material

gkad408_Supplemental_File
