# Supplementary material for: SEanalysis 2.0: a comprehensive super-enhancer regulatory network analysis tool for human and mouse
Source: Nucleic Acids Res. 2023 May 17;51(W1):W520–7. doi: 10.1093/nar/gkad408 (PMC10320134; doi:10.1093/nar/gkad408)
Supplement: gkad408_Supplemental_File [file gkad408_supplemental_file.pdf]

Supplementary Material

Gene activity score

The activity scores were calculated based on the H3K27ac signal density in gene regions using deepTools (Figure S1A). Specifically, the .bam file of each H3K27ac sample was generated from the .fastq file based on the bowtie2-Samtools pipeline. The .bam file was further converted into the .bigwig file to store the H3K27ac signal density in 50 bp bins using deepTools bamCoverage. To observe the signal intensity of the active histone H3K27ac in gene regions, we calculated the FPKM value of H3K27ac in the regions from 3 kb upstream of TSS to 3 kb downstream of TES for all genes using deepTools computeMatrix. Finally, we visualized the distribution of activity scores of SE-target genes and other genes. The specific code was as follows: "bamCoverage -b input.bam --normalizeUsing RPKM -o output.bw" and "computeMatrix scale-regions -p 10 -S output.bw -R genes.bed --beforeRegionStartLength 3000 --regionBodyLength 5000 --afterRegionStartLength 3000 --skipZeros -o output.mat.gz".

Risk SNP annotation

GWAS Catalog provides numerous risk SNPs related to diseases/traits, such as breast cancer and red blood cell count. To elucidate the role of SEs in diseases or important biological processes, we obtained information on risk SNPs from the GWAS Catalog database, which contained risk SNP locations, rsID and related diseases/traits, and so forth. These risk SNP were further filtered to delete SNPs lacking the location information.

Most SEs are composed of multiple constituent enhancers. To annotate these risk SNPs to SEs, we compared genomic positions between SNPs and constituent enhancers of SEs using BEDTools (Figure S1B). The risk SNPs were annotated to an SE region if the SNP occurred within any of the constituent enhancers of the SE. These risk SNPs occurring in between constituent enhancers or outside of SE were discarded. To link SEs to diseases/traits, we further calculated the number of annotated risk SNPs related to each disease/trait for each sample.

Sequence conservation

To calculate the sequence conservation of each SE region, we first downloaded "hg38.phastCons100way.bw" and "mm10.60way.phastCons.bw" from UCSC browser. Among these, the "hg38.phastCons100way.bw" was downloaded from 'https://hgdownload.soe.ucsc.edu/goldenPath/hg38/phastCons100way/', which contains phastCons scores for multiple alignments of 99 vertebrate genomes to the human genome. The "mm10.60way.phastCons.bw" was downloaded from 'https://hgdownload.cse.ucsc.edu/goldenPath/mm10/phastCons60way/', which contains phastCons scores for multiple alignments of 59 vertebrate genomes to the mouse genome. Then, we used the bigwigAverageOverBed tool to calculate the mean phastCons score within SE region. In the output table, we extracted the fifth column "mean0" as conservation of each SE, which is average over bases with non-covered bases counting as zeroes.

Table S1. SEanalysis 2.0 content compared with previous version of SEanalysis 1.0

Data/Analysis functions	SEanalysis 1.0	SEanalysis 2.0
Species	Human	Human and **Mouse**
Sample	542	**2670**
Super-enhancer	331,601	**1,717,744**
TF ChIP-seq samples	5042	**10,710 and 1051**
TF motifs	3279	**3680 and 742**
Risk SNP	NO	**YES**
TF regulatory analysis	NO	**NEW**
Sample comparative analysis	NO	**NEW**
Pathway downstream analysis	YES	YES
Upstream regulatory analysis	YES	YES
Genomic region annotation	YES	YES

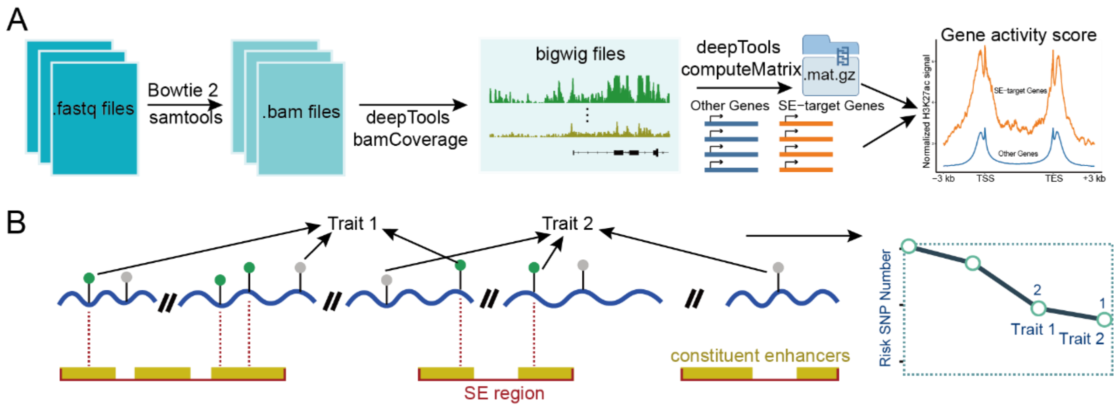

Figure S1. Gene activity score calculation and risk SNP annotation. (A) deepTools was used to calculate gene activity scores. (B) Risk SNPs were annotated to SE regions. The number of annotated risk SNPs in each trait was also calculated.

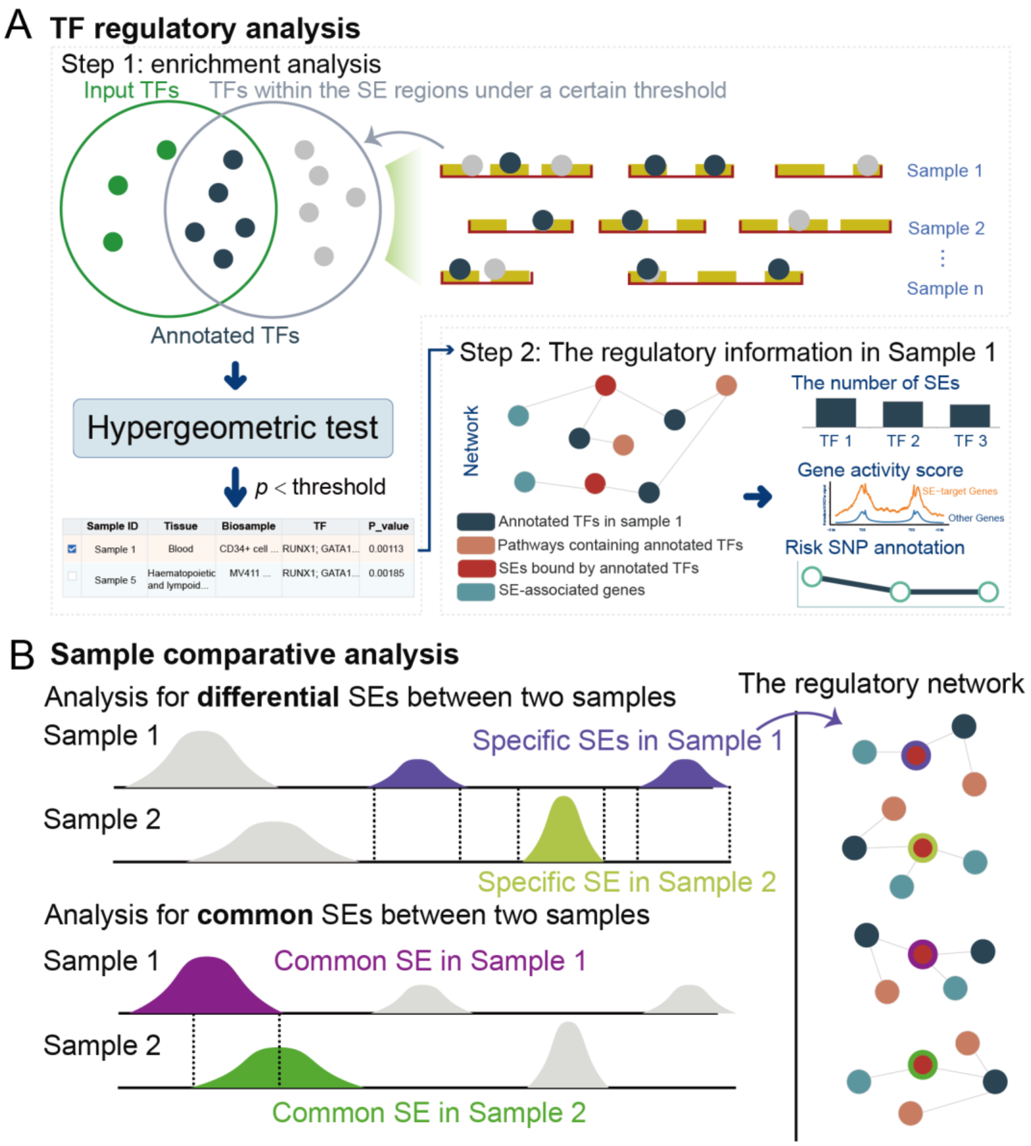

Figure S2. Analysis workflow of new functions in SEanalysis 2.0. (A) Analysis workflow of TF regulatory analysis. (B) Analysis workflow of sample comparative analysis.
